# Low humidity enhances Zika virus infection and dissemination in *Aedes aegypti* mosquitoes

**DOI:** 10.1128/msphere.00401-24

**Published:** 2024-08-02

**Authors:** Angel Elma I. Abu, Margaret Becker, Anastasia Accoti, Massamba Sylla, Laura B. Dickson

**Affiliations:** 1 Department of Microbiology and Immunology, University of Texas Medical Branch, Galveston, Texas, USA; 2 Laboratory Vectors and Parasites, Department of Livestock Sciences and Techniques, Sine Saloum University El Hadji Ibrahima NIASS, Kaffrine, Senegal; 3 Center for Vector-borne and Zoonotic Diseases, University of Texas Medical Branch, Galveston, Texas, USA; 4 The West African Center for Emerging Infectious Diseases, Centers for Research in Emerging Infectious Diseases, Galveston, Texas, USA; 5 Institute for Human Infections and Immunity, University of Texas Medical Branch, Galveston, Texas, USA; U.S. Centers for Disease Control and Prevention, Atlanta, Georgia, USA

**Keywords:** climate change, mosquito, arbovirus, Zika virus

## Abstract

As climate change alters Earth’s biomes, it is expected the transmission dynamics of mosquito-borne viruses will change. While the effects of temperature changes on mosquito-virus interactions and the spread of the pathogens have been elucidated over the last decade, the impact of relative humidity changes is still relatively unknown. To overcome this knowledge gap, we exposed *Aedes aegypti* females to various humidity conditions. We measured different components of vectorial capacity such as survival, blood-feeding rates, and changes in infection and dissemination of Zika virus. Survival decreased as the humidity level decreased, while infection rates increased as the humidity level decreased. Alternatively, blood feeding rates and disseminated infection rates peaked at the intermediate 50% relative humidity treatment but were the same in the 30% and 80% relative humidity treatments. These results provide empirical evidence that *Ae. aegypti* exposure to low humidity can enhance Zika virus infection in the mosquito, which has important implications in predicting how climate change will impact mosquito-borne viruses.

**IMPORTANCE:**

Viruses transmitted by mosquitoes to humans are a major public health burden and are expected to increase under climate change. While we know that temperature is an important driver of variation in arbovirus replication in the mosquito, very little is known about how other relevant climate variables such as humidity will influence the interaction between mosquitoes and the viruses they transmit. Given the variability in humidity across environments, and the predicted changes in humidity under climate change, it is imperative that we also study the impact that it has on mosquito infection and transmission of arboviruses.

## OBSERVATION

Arthropod-borne viruses (arboviruses) such as Zika (ZIKV), dengue (DENV), and chikungunya (CHIKV) viruses transmitted by the mosquito *Aedes aegypti* pose a major threat to public health (1, 2). Climate models predict that as global temperatures rise, the risk for arboviral disease will increase and that arboviruses will become a greater threat globally, and specifically in Africa (3, 4). Global expansion of Zika (5, 6) and year-round transmission potential from *Ae*. *aegypti* is likely to expand particularly in South Asia and sub-Saharan Africa (7). While most predictions about the impact of climate change on vector-borne disease transmission are focused on temperature, little is known about how other climate variables will impact vector-borne disease dynamics. A climate variable tightly linked to temperature is humidity (8), and the dehydration status in mosquitoes is important for physiology (9, 10).

Water loss, or dehydration, has been linked to phenotypes impacting vectorial capacity. It has also been directly associated with changes in mosquito behavior, leading to an increase in blood-feeding rate and potential increases in transmission of West Nile virus (WNV) (11–13). Additionally, water loss has been connected to decreased survival and oviposition (14).

A single study has empirically measured the effect of low humidity on infection (15). In this study, *Aedes aegypti* mosquitoes were exposed to either 35% relative humidity (RH), 75% RH, or 80% RH for 18 hours prior to the infectious bloodmeal containing Mayaro virus (MAYV). Manzano-Alvarez et al. reported that there were no significant differences in infection, dissemination, or transmission rates (rate of MAYV infection in saliva) at either 7 days post-infection (dpi) or 14 dpi between the treatments, and there was also no significant difference in survival. However, they did observe that the blood-feeding rate of the 75% group was significantly higher than the 35% group and control; the latter two did not have a statistically significant difference between them.

**Fig 1:**
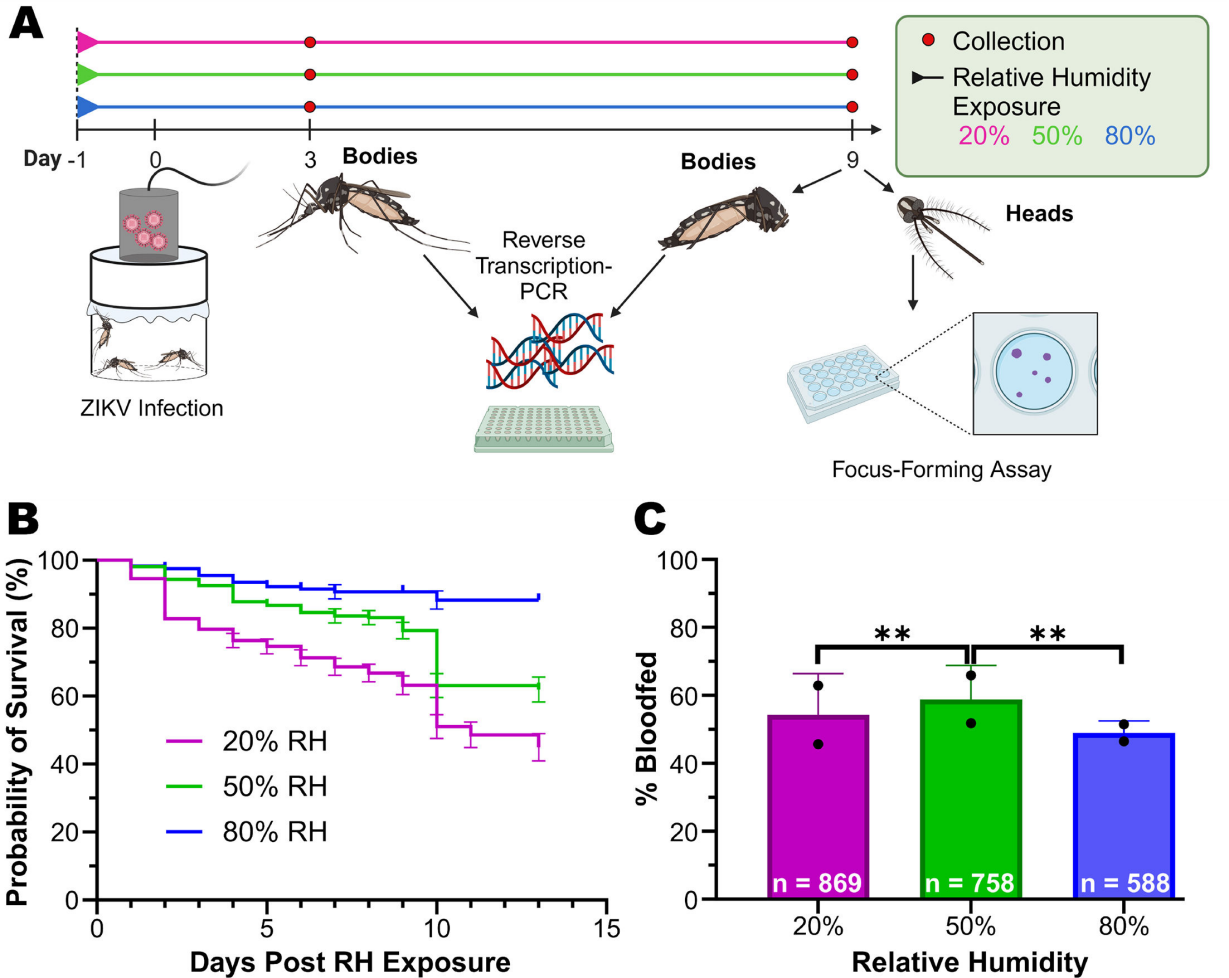
Low humidity impacts survival probability and blood-feeding rate. (**A**) F12 *Ae. aegypt*i collected in Thíes, Senegal (16), were exposed to 20%, 50%, or 80% RH treatments 24 hours prior to the infectious blood feeding and maintained at those humidity levels for the duration of the experiment. Experiments were conducted in two biological replicates. On day 0, mosquitoes were fed a blood meal infected with the Zika virus (Cambodia FSS 13025 isolate) at a titer of 3.4 × 10^7^ FFU/mL. Collections were made on days 3 and 9 to determine the infection and dissemination rates. Infection rates were determined in bodies by RT-PCR of the bodies 3 and 9 dpi, and dissemination rates and titers were determined in heads 9 dpi through a focus-forming assay. (**B**) Probability of survival over a span of 13 dpi bloodmeal for each treatment. A log-rank (Mantel-Cox) test was performed as a mean of two replicates (*P*-value < 0.0001) with sample sizes of 433 individuals from the 20% RH treatment, 427 from the 50% RH treatment, and 289 from the 80% RH treatment. (**C**) Blood-feeding rates were determined by the number of fully engorged mosquitoes divided by the total number that was offered the bloodmeal. The results are presented as a mean ($\bar{x}$) proportion bloodfed of two replicates from sample sizes of 869 (20% RH), 758 (50% RH), and 588 (80% RH). The effect of overall humidity on blood-feeding rates and differences between pairwise comparisons were determined by a two-tailed chi-squared analysis. Overall humidity *P*-value = 0.0098, 20% vs 50% *P*-value = 0.0087, 20% vs 80% *P*-value = 0.7996, 50% vs 80% *P*-value = 0.0088.

To test the impact of low humidity on various phenotypes related to vectorial capacity, *Ae. aegypti* mosquitoes collected from the city of Thíes in Senegal (16) were exposed to Zika virus under three relative humidity ranges, 20% ± 10%, 50% ± 10%, or 80% ± 10% at 28°C for 24 hours prior to the infectious bloodmeal and the duration of infection. We measured survival, blood-feeding, body infection rates, and disseminated infection rates, as well as dissemination titers (Fig. 1A). Survival rates were defined by the daily probability of death and blood-feeding rates were defined as the total number of mosquitoes that were fully engorged on blood divided by the total number of mosquitoes that were offered a blood meal for each treatment. Body infection and disseminated infection rates were the proportion of bodies or heads, respectively, that tested positive for ZIKV infection out of all fully engorged mosquitoes. Dissemination titers were the number of formula-forming units (FFU) present in the heads of infected mosquitoes. Detection of ZIKV RNA (reverse transcription-polymerase chain reaction [RT-PCR]) and infectious particles (FFU/mL) was done as previously described (17). Mean ($\bar{x}$) infection rate values represent the mean infection rate of two independent replicates. Detailed materials and methods are included in Fig. S2. Statistics are summarized in Table S1 and raw data tables are present in Table S2.

The relative humidity and temperature were monitored by digital hygrometers (VWR and Hobo) for the duration of the study to ensure expected environmental conditions. Following exposure to low humidity, mosquitoes exhibited lower survival probabilities as the humidity decreased (Mantel-Cox, *P*-value < 0.0001) (Fig. 1B). Blood-feeding rate was also observed as a function of humidity (chi-square on GLM, humidity: *P*-value = 0.0098). A significant effect of replicate was observed (replicate: *P*-value = 1.18 × 10^7^) driven by higher overall feeding rates in replicate two compared to replicate one. Importantly, the interaction between humidity treatment and replicate was not significant (humidity × replicate: *P*-value = 0.0821) indicating the effect of humidity treatment on feeding rates was consistent between replicates. The 20% ($\bar{x}$ = 54.5% bloodfed) and 80% ($\bar{x}$ = 49.2% bloodfed) RH treatments did not have significantly different blood-feeding rates from each other (chi-square, *P*-value = 0.7996). However, they had lower mean feeding rates compared to the 50% humidity treatment ($\bar{x}$ = 59.05% bloodfed), which was statistically significant when compared to the 20% RH (chi-square, *P*-value = 0.0087) and 80% RH treatments (chi-square, *P*-value = 0.0088) (Fig. 1C). These results were similar to the previous study on MAYV in which they also observed an increase in blood-feeding rates in their intermediate humidity level (15).

**Fig 2:**
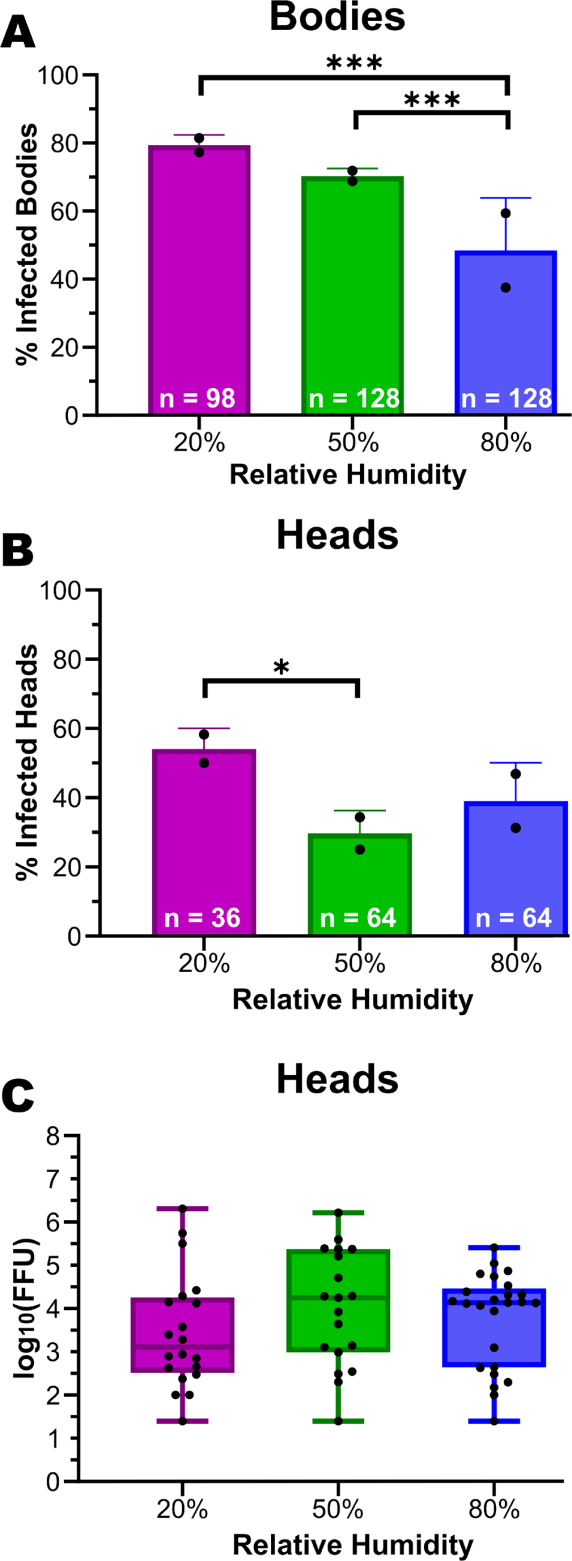
Low humidity increases infection rates and dissemination but has no impact on dissemination titers. (**A**) The mean ($\bar{x}$) percentage of infected bodies is shown for each humidity treatment. Results from individuals collected 3 and 9 dpi were combined due to having no statistically significant difference between the two collection time points. Infection rates were determined by detecting ZIKV RNA by RT-PCR. The overall impact of humidity level on body infection rates was analyzed by chi-square on a binomial logistic regression (humidity: *P*-value = 1.93 × 10^6^). Data represents two independent replicates with sample sizes of 98 (20% RH), 128 (50% RH), and 128 (80% RH). Significance between treatments was assessed through a two-tailed chi-square analysis with the following *P*-values: 20% vs 50% = 0.1135, 20% vs 80% = 0.0001, 50% vs 80% = 0.0004. (**B**) The percentage of infected heads is shown for each humidity treatment. Infection was determined in heads 9 dpi through a focus-forming assay. The percentage of infected heads was determined by the number of infected samples divided by the total number tested per treatment. Plotted values represent the mean ($\bar{x}$) of two independent replicates with sample sizes of 36 individuals from the 20% RH treatment, 64 from the 50% RH treatment, and 64 from the 80% RH treatment. The overall impact of humidity level on body infection rates was analyzed by chi-square on a binomial logistic regression (humidity: *P*-value = 0.03996). Significance between pairwise treatments was assessed through a two-tailed chi-square analysis with the following *P*-values: 20% vs 50% = 0.0109, 20% vs 80% = 0.1115, 50% vs 80% = 0.2642. (**C**) The mean viral titer was calculated as the log_10_(FFU)/mL per head. The overall impact of humidity level on the number of infectious particles in the head was calculated by two-way analysis of variance (ANOVA) on a logistic regression (humidity: *P*-value = 0.6514). Pairwise comparisons were made with paired *t*-tests with the following *P*-values: 20% vs 50% = 0.3878, 20% vs 80% = 0.1777, and 50% vs 80% = 0.3843.

To understand the impact of relative humidity on ZIKV infection in *Ae. aegypti*, adult females were harvested at 3 and 9 dpi. Overall, differences in whole-body infection rates can be explained by humidity treatment (chi-square on GLM, humidity: *P*-value = 1.93 × 10^−6^). The difference in harvest day had no impact on the effect of the RH treatment on body infection rates (humidity × dpi: *P*-value = 0.8034) (Fig. S1), so data from the two-time points were combined. In pairwise comparisons, mosquitoes maintained at 20% RH ($\bar{x}$ = 79.38% infected, *P*-value = 0.0001) and 50% RH ($\bar{x}$ = 70.32% infected, *P*-value = 0.0004) had significant increases in infection rates compared to the 80% RH treatment ($\bar{x}$ = 48.44% infected). However, there was no significant difference between the 20% and 50% RH treatments (*P*-value = 0.1135) even though infection rates trended higher in the 20% RH treatment (Fig. 2A).

To test whether low humidity increases ZIKV disseminated infection rates, the proportion of mosquitoes that developed a disseminated infection out of the total number exposed was measured by detecting infectious virus particles in the head 9 dpi by focus-forming assay. Overall, differences in disseminated infection rates can be explained by humidity (chi-square on GLM, humidity: *P*-value = 0.0400). In pairwise comparisons, mosquitoes maintained at 20% ($\bar{x}$ = 54.17% disseminated, *P*-value = 0.1115) and 50% ($\bar{x}$ = 29.69% disseminated, *P*-value = 0.2642) showed no significant difference in the proportion of disseminated infection compared to the 80% RH treatment ($\bar{x}$ = 39.07% disseminated). However, there were significant differences between the 20% and 50% RH treatments (*P*-value = 0.0109) (Fig. 2B) with dissemination peaking in the 50% RH treatment. Additionally, the quantity of infectious virus particles was measured in the heads to determine if low humidity exposure increased replication of the virus. The results showed a mean dissemination titer in log_10_(FFU)/mL of 3.50 in the 20% RH, 4.01 in the 50% RH, and 3.76 in the 80% RH. Humidity treatment had no impact on the number of infectious virus particles in the heads between the treatments (ANOVA, *F* = 0.4318, *P*-value = 0.4377) (Fig. 2C). Statistics are summarized in Table S1.

In contrast to Manzano-Alvarez et al. (15), we observed that mosquito infection and dissemination rates increase in response to low humidity. Perhaps conflicting results between this study and Manzano-Alvarez et al. are a result of the different genetic backgrounds of *Ae. aegypti* used different impacts of low humidity on alphaviruses vs flaviviruses or differences between the timing and duration of low humidity exposure. The highly inbred Liverpool line of *Ae. aegypti* was used in the Manzano-Alvarez et al. study, while an F12 line of *Ae. aegypti* collected in Thíes, Senegal, was used in this study (16). Given the contribution of mosquito genetics to vector competence (18) and the involvement of a desiccation-induced gene in midgut infection (19), it is possible that low humidity impacts differently across genetic backgrounds. Additionally, in Manzano-Alvarez et al. mosquitoes were exposed to their humidity treatments for only 18 hours per infectious bloodmeal at either 35% or 75% RH, while in this study the humidity treatments were maintained for the duration of viral incubation at either 20% or 50% RH. Furthermore, this study assayed different days post-infection than Manzano-Alvarez et al. (3 and 9 dpi here, and 7 and 14 dpi in Manzano-Alvarez et al.) but it is not anticipated the conclusions will change if we include another time point.

Interestingly, an increase in dissemination rates was only observed at the intermediate humidity level, but not at the lowest humidity level. Additionally, blood-feeding rates were the same between the lowest and highest humidity treatment and only increased in the intermediate humidity treatment. This data suggests that the mechanisms underlying the influence of low humidity on virus dissemination and blood feeding rates are likely complicated and perhaps different mechanisms exist for different humidity ranges.

Overall, this study demonstrates that exposure to low humidity increases ZIKV infection and dissemination rates in *Ae. aegypti*, which has important implications for the effect of climate on the transmission of arboviruses. This experimental framework paves the way for future mechanistic studies on how variation in humidity influences ZIKV infection and replication in the mosquito and highlights the need to include this important climate variable in future work. Given predicted changes in humidity under climate change, it is important to study the impact of these changes on vector behavior and virus transmission.

## SUPPLEMENTAL MATERIAL

Figure S1 msphere.00401-24-s0001.tiff Infection rate by day.

Figure S2 msphere.00401-24-s0002.pdf Supplemental materials and methods.

Figure legend msphere.00401-24-s0003.pdf Fig. S1 legend.

Table S1 msphere.00401-24-s0004.pdf Summary statistics.

Table S2 msphere.00401-24-s0005.xlsx Raw data.

Table legend msphere.00401-24-s0006.pdf Table S2 legend.

ASM does not own the copyrights to Supplemental Material that may be linked to, or accessed through, an article. The authors have granted ASM a non-exclusive, world-wide license to publish the Supplemental Material files. Please contact the corresponding author directly for reuse.
