# Supplementary figures and images for: Low humidity enhances Zika virus infection and dissemination in Aedes aegypti mosquitoes
Source: mSphere. 2024 Aug 2;9(8):e00401-24. doi: 10.1128/msphere.00401-24 (PMC11351097; doi:10.1128/msphere.00401-24)

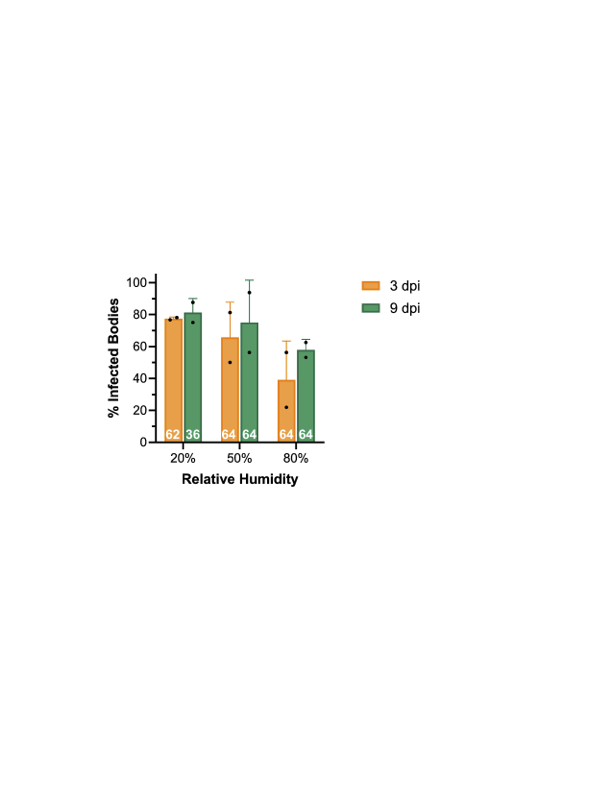

Supplement: Figure S1 — Infection rate by day. [file msphere.00401-24-s0001.tiff]
