# Supplementary material for: Low humidity enhances Zika virus infection and dissemination in Aedes aegypti mosquitoes
Source: mSphere. 2024 Aug 2;9(8):e00401-24. doi: 10.1128/msphere.00401-24 (PMC11351097; doi:10.1128/msphere.00401-24)
Supplement: Figure S2 — Supplemental materials and methods. [file msphere.00401-24-s0002.pdf]

**Supplemental Figure 2: Materials and Methods**

**Mosquito rearing and preparation**

*Aedes aegypti* colonies were sourced from a natural population sampled from the city of Thíes, Senegal using ovitraps. The mosquitoes used in these experiments were generation 11 mosquitoes reared under standard insectary conditions (28˚C and 80%±10% relative humidity with 12h:12h light dark cycle). Eggs were hatched in deionized water and larvae were fed fish food (Pentair AES). Adults were held in cages with ad libitum access to a 10% sucrose solution until needed. 4-5 days following emergence, 75 female mosquitoes were sorted into 1-pint cylindrical paper cups covered with netting prior to low humidity exposure.

**Desiccation treatment**

20 cups of 75 *Ae. aegypti* were placed in 18 1/8 in x 12 1/4 in x 7 in plastic bins (Sterilite) divided between three treatments. Desiccation was achieved by placing 6 gram bags of montmorillonite clay (Grainger) in the containers to reach the desired relative humidity levels of 20% or 50%. The 80% RH was controlled with a damp sponge. The humidity in each container was measured constantly for the duration of the experiment with digital hygrometers (VWR TraceableGO Bluetooth Datalogging Temperature and Humidity Meter and HOBO UX100-023). Disproportionate numbers of mosquitoes were exposed to each treatment to ensure that enough individuals survived for each collection. As such, 9 cups were placed in the 20% treatment, 7 cups in the 50% treatment, and 4 cups in the 80% RH treatment. Mosquitoes were exposed to the relative humidity treatments for 24 hours prior to the infectious blood feeding and kept at the same humidity levels until collections at two timepoints: three and nine days post infection. Immediately prior to blood-feeding, the number of dead in each treatment was recorded. We were unable to maintain consistent humidity levels if the mosquitoes had constant

access to wet sugar pads. To mitigate this, mosquitoes were given access to a 10% sucrose solution for 20 minutes once a day for the duration of the experiment.

**Mosquito infections**

Infections were conducted using the C6/36 culture-grown P2 Zika virus Cambodia isolate (FSS 13025) received from the World Reference Center for Emerging Viruses and Arboviruses at UTMB (WRCEVA). The virus stock was diluted in DMEM media (Gibco) supplemented with 1.5% fetal bovine serum (FBS) (R&D Systems), 1% penicillin-streptomycin (Gibco), and 1% antibiotic-antimycotic (Life Technologies), and supplemented with 60 µL of 100 mM ATP and 30 µL of 7.5% sodium bicarbonate to a final volume of 3 mL. Mosquitoes were fed a dose of $3.4 \times 10^7$ FFU/mL at normal insectary conditions for 15 minutes. Fully engorged mosquitoes were sorted and counted to determine the blood-feeding rate.

**Virus detection**

Detection of Virus in Bodies by RT-PCR

At days three and nine post infection, the heads were separated from their bodies and both were stored at -80°C until further processing. Bodies were homogenized in 300 µL of a formulated RNA extraction buffer (10 mM Tris HCl, 50 mM NaCl, 1.25 mM EDTA, 0.35 g/L proteinase K) at 6800 rpm for three 20 second cycles with 30 second pauses between cycles. Total RNA was converted into complementary DNA (cDNA) using M-MLV reverse transcriptase (Invitrogen) and random hexamers in the following reaction: 10 min at 25°C, 50 min at 37°C, and 15 min at 70°C. The cDNA was amplified by PCR in a reaction containing 1X DreamTaq DNA polymerase (Thermo Fisher Scientific) and ZIKV primers (forward: 5'-GTATGGAATGGAGATAAGGCCCA-3', and reverse: 5'-ACCAGCACTGCCATTGATGTGC-3'). Cycling conditions were as follows, 2 min at 95°C, followed by 35 cycles of 30 s at 95°C, 30 s at

60°C, and 30 s at 72°C with a final extension step of 7 min at 72°C. Amplicons were visualized on a 2% agarose gel.

Detection of Virus in Heads by Focus Forming Assay

Heads from mosquitoes harvested nine days post infection were homogenized in 200 µL DMEM media (Gibco) supplemented with 1.5% fetal bovine serum (FBS), 1% penicillin-streptomycin (Gibco), and 1% antibiotic-antimycotic (Life Technologies). Vero cells were seeded in 24-well plates and incubated for 48 hours to reach confluency. Each well was inoculated with 200 µl of head homogenate in 10-fold dilutions (from $10^1$ to $10^4$) and incubated at 37°C (5% $CO_2$) for 1 hour, rocking every 15 minutes. Infected cells were overlaid with α-MEM media supplemented with 1.25% carboxymethyl cellulose, 5% FBS, and 1% Pen-Strep. After three days of incubation at 37°C, infected cells were fixed with 10% formalin for at least 1 hour and were washed three times in 1X PBS. Plates were then rocked with 500 µl of blocking solution (5% w/v non-fat powdered milk in 1X PBS) per well for 30 minutes. The blocking solution was discarded and replaced with 200 µl of ZIKV primary antibody diluted 1:1000 in blocking solution (obtained from WRCEVA). The plates were then placed on a rocker overnight. The primary antibody solution was discarded, and plates were washed three times with 1X PBS after which 200 µl of secondary antibody (peroxidase-labeled goat anti-mouse IgG human serum KPL-474-1806) solution (diluted 1:2000 in blocking solution) was added to each well. Plates were placed on plate rocker for 1 hour. The secondary antibody solution was discarded, and plates were again washed three times with 1X PBS. To develop visible foci, 100µl of TrueBlue peroxidase substrate (KPL 5510-0050) was added to each well, and plates were placed on the plate rocker until foci could be seen, around 5 minutes. Plates were washed with deionized water and the foci were counted. Focus-forming units were $Log_{10}$ transformed to represent the concentration of infectious ZIKV particles detected in Ae. aegypti heads.

**Statistical Analysis of Survival, Blood-feeding, Infection, and Dissemination Rates**

Statistical analysis was calculated using GraphPad Prism software version 10 and R. Statistical significance of survival probabilities was analyzed using a log-rank Mantel-Cox test. Blood-feeding rate, infection rate, and dissemination rate and titers were analyzed by two-way ANOVA on the linear regression; significance between treatments for these tests was assessed through a two-tailed Chi-square analysis. Summary of statistical tests can be found in Supplemental Table 1 and raw data for all experiments is available in Supplemental Table 2.

R code to perform Chi-square on GLM:

```
model <- glm(Status ~ Humidity*dpi*Replicate, family=binomial(link='logit'), data)

anova(model, test="Chisq")
```

R code to perform two-way ANOVA on logistic regression:

```
test <- lm(formula = `log(FFU)` ~ Humidity*Replicate, data)

Anova(test, type=2)
```
