# Supplementary material for: Low humidity enhances Zika virus infection and dissemination in Aedes aegypti mosquitoes
Source: mSphere. 2024 Aug 2;9(8):e00401-24. doi: 10.1128/msphere.00401-24 (PMC11351097; doi:10.1128/msphere.00401-24)
Supplement: Figure legend — Fig. S1 legend. [file msphere.00401-24-s0003.pdf]

Supplemental Figure 1: Relative humidity treatment did not affect the rate of infection between three and nine dpi The percentage of infected bodies at three and nine dpi is shown for each humidity treatment. Mosquito bodies were collected at three and nine dpi to determine infection rate. Infection rates were determined by detecting ZIKV RNA by RT-PCR. Infection rates were analyzed by Chi-square on a binomial logistic regression linear regression of the data to test the effects of treatment on harvest day. While there was consistently a higher infection rate nine dpi there was no interaction between day of harvest and RH treatment when it came to infection (Humidity * dpi: p-value = 0.8034). Summary of statistical tests can be found in Supplemental Table 1 and raw data for all experiments is available in Supplemental Table 2.
