# Supplementary material for: Low humidity enhances Zika virus infection and dissemination in Aedes aegypti mosquitoes
Source: mSphere. 2024 Aug 2;9(8):e00401-24. doi: 10.1128/msphere.00401-24 (PMC11351097; doi:10.1128/msphere.00401-24)
Supplement: Table S1 — Summary statistics. [file msphere.00401-24-s0004.pdf]

Supplemental Table 1: Summary of statistical analyses

Survival Probability							
	Df	Chi-squared	P-value	*Pairwise*	Df	Chi-squared	P-value
Treatment	2	72.27	0.0001	20% vs 50%	1	25.3	0.0001
				20% vs. 80%	1	62.18	0.0001
				50% vs. 80%	1	17.12	0.0001
**Blood-feeding Rate**							
	Df	Deviance Residual	P-value	*Pairwise*	Df	Chi-squared	P-value
Treatment	2	9.2606	0.009752	20% vs 50%	1	6.875	0.0087
Replicate	1	28.0608	1.18E-07	20% vs. 80%	1	0.064	0.7996
Treatment:Replicate	2	4.9977	0.082179	50% vs. 80%	1	6.862	0.0088
**Infection Rate**							
	Df	Deviance Residual	P-value	*Pairwise*	Df	Chi-squared	P-value
Treatment	2	26.32	1.93E-06	20% vs 50%	1	2.505	0.1135
Replicate	1	1.5661	0.21078	20% vs. 80%	1	22.854	0.0001
Treatment:Replicate	2	5.2155	0.0737	50% vs. 80%	1	12.696	0.0004
**Dissemination Rate**							
	Df	Deviance Residual	P-value	*Pairwise*	Df	Chi-squared	P-value
Treatment	2	6.4397	0.03996	20% vs 50%	1	6.481	0.0109
Replicate	1	0.3044	0.58111	20% vs. 80%	1	2.532	0.1115
Treatment:Replicate	2	2.2454	0.32541	50% vs. 80%	1	1.247	0.2642
**Dissemination Titers**							
	Df	F-value	P-value	*Pairwise*	Df		P-value
Treatment	2	0.4318	0.65138	20% vs 50%	1		0.3878
Replicate	1	3.237	0.0772	20% vs. 80%	1		0.1777
Treatment:Replicate	2	2.7105	0.07493	50% vs. 80%	1		0.3843

Statistical analysis was conducted using GraphPad Prism software version 10 and R. Survival probabilities were analyzed using a log-rank (mantel-cox) test for both overall impact of treatment and pairwise comparisons. Overall impact of treatment and replicate were analyzed by Chi-square on a binomial logistic regression for each test except for dissemination titers, which were analyzed by two-way ANOVA on a logistic regression. Pairwise comparisons for dissemination titers were done by t-tests (Kolmogorov-Smirnov). Pairwise comparisons for all other tests were done with two-tailed Chi-squared tests on 2x2 contingency tables.
