# Supplementary material for: Low humidity enhances Zika virus infection and dissemination in Aedes aegypti mosquitoes
Source: mSphere. 2024 Aug 2;9(8):e00401-24. doi: 10.1128/msphere.00401-24 (PMC11351097; doi:10.1128/msphere.00401-24)
Supplement: Table legend — Table S2 legend. [file msphere.00401-24-s0006.pdf]

Supplemental Table 2: Raw Data

Raw data for each assay separated by tab.
